# Changes in Adenylate Nucleotides Concentration and Na^+^, K^+^-ATPase Activities in Erythrocytes of Horses in Function of Breed and Sex

**DOI:** 10.4061/2010/987309

**Published:** 2009-11-11

**Authors:** Maria Suska, Ewa Skotnicka

**Affiliations:** Department of Physiology, Faculty of Life Sciences, University of Szczecin, Piastów 40 B, av, bl. 6, 71-065 Szczecin, Poland

## Abstract

The aim of this study was to examine the relationships between the concentrations of ATP, ADP, AMP (HPLC methods), total nucleotide pool (TAN), adenylate energy charge (AEC) and Na^+^, K^+^-ATPase erythrocytic activities (by Choi's method) of horses as a function of breed and sex. The studies were conducted on 54 horses (stallions and mares) of different constitution types: breathing constitution (Wielkopolska and Hanoverian breed) and digestive constitution (Ardenian breed). Horse erythrocytes, independently of examined breed, present low ATP concentration in comparison to other mammal species while retaining relatively high AEC. Erythrocytes of breathing constitution type horses appear to have a more intensive glucose metabolism and a more efficient energetic metabolism when compared to digestive constitution type horses. The conclusions may be proven by significantly higher ATP concentration, higher TAN and significantly higher AEC in breathing constitution type horses compared to the digestive constitution type. Sex does not significantly influence adenine nucleotides concentration in the erythrocytes of the examined horses, however, stallions have slightly higher values in comparison to mares. A positive correlation was found between Na^+^, K^+^, -ATPase activity, ATP, ADP and AMP concentration and TAN in Wielkopolska and Ardenian breeds, which was not confirmed for the Hanoverian breed.

## 1. Introduction

The horse *(Equus caballus L.) *is the only mammal that went through evolution in the steppes. Lacking an active protection mechanism against predators, escape is the only way to protect its life and thus to guarantee the survival of the species. To allow for an intensive run, during which oxygen consumption can be up to 35 times higher than in resting tomes, both the anatomical structure and the overall metabolism of horses have been optimized during evolution to favour energy supply to the muscles. Contemporary horses, selected by man according to his needs or likes, did not loose their elementary cell characteristic of quick energy delivery and high energy consumption. This also holds true for erythrocytes, which play a key physiological function in oxygen binding and transport to the tissues.

 The study of purine nucleotide metabolism is very important for the understanding of disruptions in energy metabolism as the purine nucleotides participate in most energy-requiring metabolic reactions and act as coenzymes [1, 2]. Characteristic compounds in the erythrocyte energy metabolism are the adenylate nucleotides (ATP, ADP, AMP) which undergo reversible changes in glycolysis and in pentose phosphate pathway [3, 4]. They are involved in the control of the correct shape of red blood cells, active transport through the membrane, phosphorylation of proteins and phospholipids, and the synthesis of glutathione and pyridine coenzymes Moreover, ATP plays a significant role in transferring energy within the cell, diffusing from where it is produced to where it is utilized. ATP in erythrocytes is utilized in ion transport processes that account for almost 30% of the total ATP consumption. Na^+^ and K^+^ transport utilizes a lot of ATP, while Ca^2+^ transport requires markedly less energy. There is no consensus in literature on the dependence of Na^+^, K^+^-ATPase (EC 3.6.1.37) and Ca^+^-ATPase (EC 3.6.1.3) on ATP. For this reason we also attempted to find out the relations between the ATP concentration and the activity of Na^+^, K^+^-ATPase (EC 3.6.1.37) [5–9]. Mathematic modelling suggests that during changes in ATP-consuming processes, adenylate metabolism may regulate the adenylate energy charge (AEC) of the cell [10].

 The rate of glucose metabolism and the concentration of metabolites of the glycolytic pathway in the erythrocytes of various species of mammals, as well as the concentration of the adenylate nucleotides, are genetically determined [11] and also depend on species and age [12–14]. Different exogenous (heavy metal ion influence; such as fluorine, lead) and endogenous (physical effort, pregnancy, morbidity such as diabetes) factors may also influence these values [15–18].

 The aim of this study was to compare the erythrocyte energy metabolism in stallions and mares of three horse breeds of different constitution and different functional characteristics (Wielkopolska breed, Hanoverian breed, Ardenian breed). The study was to determine the relationships between the ATP, ADP, AMP and Na^+^, K^+^-ATPase activities on one hand, and the level of TAN and AEC values on the other, in the erythrocytes as a function of breed and sex. 

## 2. Materials and Methods

### 2.1. Animals and Management Practices

The studies were conducted for 54 clinically healthy horses aged 3-4 years of different constitution type: Wielkopolska breed (W: n = 8 stallions and w: n = 8 mares), Hanoverian breed (H: n = 8 stallions and h: n = 10 mares), Ardenian breed ( A: n = 10 stallions and a: i = 10 mares). The horses came from environmentally clean areas, were not in training and were fed and treated equally. The animals had a standard diet with respect to their age. The horses did not show any sign of particular stress during blood sampling. All studies were approved by the Local Ethics Committee.

### 2.2. Blood Sampling

In order to eliminate the influence of physical strain and circadian changes on some biochemical indices, 2 mL of blood per heparinized test tube (250 IU heparine from Polfa Poland) was taken from the external jugular vein early in the morning before feeding. The blood was delivered to the laboratory in ice flasks and immediately analyzed.

#### 2.2.1. HPLC Separation of Purine

 (1) Chemicals. Purines used as chromatographic standards were obtained from Sigma-Aldrich. HPLC grade acetonitrile was obtained from Merck. HPLC grade potassium dihydrogen orthophosphate, potassium chloride and tripotassium orthophosphate were obtained from Fluka Chemie GmbH. HPLC grade water was prepared using a Milli-Q (Millipore purification system). All HPLC solvents were filtered through 0.22 *μ*m nylon filters (Supelco) before use.

(2) *Instrumentation*. For the HPLC analysis, a Hewlett Packard chromatographic system was used. The HP series 1100 chromatographic system consisted of a quaternary pump system with a degasser and a continuous seal wash option (G1311A), a variable-wavelength detector (G1314) and a thermostatted column compartment (G1316A). The analytical column (100 × 4.6 mm LC) was packed with 18.3 *μ*m Hypersil BDS-C (Hewlett Packard). Samples were introduced using a Rheodyne 7725 injection valve equipped with a 20 *μ*m loop. Sample peaks were integrated and quantified using an HPLC chromatography data system operating on Chemstation Software for Windows 98 (Hewlett Packard).

(3) *HPLC separation of adenine nucleotides. *The samples (500 *μ*L) were deproteinized with 500 *μ*L of 1.3 M perchloric acid in 1.5 mL Eppendorf tubes. Extract mixtures were centrifuged (16 000 g for 10 minutes, at 4°C). The supernatant (600 Jll) was neutralized with approximately 60–90 *μ*L of 3 M potassium orthophosphate solution (pH range of the sample at 6.3). The neutralized extract was centrifuged again and filtered through a nylon filter of 0.22 *μ*m. The obtained clear filtrate was used directly for the HPLC assay or stored at −80°C.

(4) *Purine nucleotide determination. *Concentrations of purine nucleotides: ATP, ADP, and AMP were measured by the HPLC method of Smolenski et al. [19]. Sample aliquots (100 *μ*L) were injected into the chromatograph column and the nucleotides were separated using a linear phosphate buffer gradient system at the flow rate of 0.666 mL/min (buffer A: 150 mM KH_2_PO_4_, 150 mM KCI adjusted to pH 6.0 with K_2_HPO_4_; buffer B: 15% v/v solution of acetonitrile in buffer A). Peaks were detected by absorption measurements at 254 nm. The composition of the mobile phase was controlled by a low-pressure gradient mixing device. The cycle time was 12.8 minutes between injections. The analytical column was maintained at constant temperature of 20.5°C. The adenine nucleotide concentrations (*μ*M in the whole blood) were calculated according to factors given in the method of Smolenski et al. [19]. Then the adenine nucleotide concentrations in the erythrocytes were calculated using the value of the packed cell volume (PCV).

The total adenylate nucleotides (TAN) and the adenylate energy charge (AEC) were calculated according to the formulas:

(1) $$\begin{array}{c} \text{TAN}=\left[\text{ATP}\right]+\left[\text{ADP}\right]+\left[\text{AMP}\right]\\ \text{AEC}=\frac{1}{2}*\frac{\left[\text{ADP}\right]+2\left[\text{ATP}\right]}{\left[\text{ATP}\right]+\left[\text{ADP}\right]+\left[\text{AMP}\right]}. \end{array}$$

## 3. Measurements of ATPase Activities

The Na^+^, K^+^-ATPase activities were determined on erythrocyte plasma membranes by Choi's method [20]. For the determination of membrane ATPase, aliquots of a 25 *μ*L membrane suspension, containing 25–50 *μ*g of protein, were preincubated at 37°C for 5 minutes in the following media: 20 mM KCl; 100 mM NaCl; 0.5 mM EGTA; 30 mM histidine-imidasole buffer (pH 7.0); 0.5 mM MgCl_2_. The enzymatic reaction was started by adding 2 mM ATP. After 30 minutes of incubation at 37°C, the reaction was stopped by adding 1 mL of ice cold 20% trichloric acid. Then the mixture was centrifuged at 20 000 G for 2 minutes and the inorganic phosphate released into the supernatant was measured, according to the Goldenberg and Fernandez method [21]. Enzyme activity is expressed as of nmol of *P*_*i*_ per mg membrane proteins per 60 minutes determined according to Lowry et al. [22].

### 3.1. Statistical Evaluation

The results are given as mean values $\left(\overset{®}{x}\right)$ and standard deviations (SD). The statistical significance of differences among the groups was determined with the monofactor analysis of variance (ANOVA) using the evaluation of statistical significance of differences for many means and NIR Tukey's test (paired design). The Pearson's correlation coefficient was determined in all the groups (according to the Statistica PL software) for all adenylate nucleotide, TAN and ATPase activity, as well as the value of the AEC. The regression lines with marked values of correlation coefficients [*r*] were drawn for the significant relationships within the groups.

## 4. Results

Mean values and standard deviations determined for the adenine nucleotides concentrations, nucleotides pool and adenylate energy charge in the erythrocytes and in chosen haematological indicators (RBC, Hb) of stallions: Wielkopolska breed (W), Hanoverian breed (H), Ardenian breed (A) were gathered in Table 1, and for mares (w, h, a) in Table 2. 

We determined statistically significant differences in ATP concentrations in stallions and mares of all the breeds, respectively, at *P* < .05 and *P* < .01 (Tables 1 and 2). The highest ATP concentration was determined in Wielkopolska stallions (178.3 ± 12.5 *μ*M_*Μ*RBC_) and mares (175.4 ± 9.6 *μ*M_*Μ*RBC_). The lowest ATP values were found in the Ardenian horses, both stallions (148.7 ± 12.5 *μ*M_*Μ*RBC_), and mares (147.3 ± 21.2 *μ*M_*Μ*RBC_), while average values were found in the Hanoverian breed (stallions-155.1 ± 11.3 *μ*M_*Μ*RBC_, mares-150.4 ± 21.5 *μ*M_*Μ*RBC_). Wielkopolska stallions have significantly higher ATP concentrations in comparison to Hanoverian (*P* < .05) and Ardenian breed (*P* < .01). The difference of ATP concentration between Hanoverian and Ardenian breeds was not statistically significant. Slightly higher ATP erythrocyte concentrations were found in stallions in comparison to mares regardless of breed (Tables 1 and 2). 

 ADP and AMP concentrations in the erythrocytes of Hanoverian stallions were the lowest among the examined breeds. However, significant differences for the determined parameters were found between Hanoverian and Ardenian stallions *P* < .01 (Table 1).

Nucleotides pool values (TAN) in the erythrocytes of stallions and mares of Wielkopolska breed have a higher value in comparison to Hanoverian and Ardenian breeds (stallions-212.1 ± 14.7 *μ*M_*Μ*RBC_, mares-209.3 ± 10.7 *μ*M_*Μ*RBC_). The differences were statistically significant at *P* < .05 (Table 1). Stallions of all the examined breeds have higher adenine nucleotides pool (TAN) in comparison to mares. However, in none of the examined groups statistically significant differences were found. No significant differences in adenylate energy charge (AEC) were found between stallions and mares (Tables 1 and 2). 

A significant intra-group positive correlation between the ATP erythrocyte concentration and the nucleotides pool TAN was found in stallions and mares of all three breeds. Statistically significant correlation was found between ATP and adenylate energy charge (AEC) values in the Hanoverian and Ardenian, whereas in the Wielkopolska mares the correlation coefficient value although significant, was the lowest (Table 3). 

The lowest Na^+^, K^+^-ATPase activity of erythrocytes was found in the Ardenian horses, both stallions (0.22 ± 0.05 *μ*M *P*_*i*_/mg protein/h) and mares (0.19±0.02 *μ*M *P*_*i*_/mg protein/h). Both Wielkopolska and Hanoverian stallions presented similar activity of the examined enzyme, respectively, 0.34 ± 0.06 *μ*M *P*_*i*_/mg protein/h and 0.34 ± 0.01 *μ*M *P*_*i*_/mg protein/h; the enzyme activity in Wielkopolska mares was (0.28 ± 0.02 *μ*M *P*_*i*_/mg protein/h) and in Hanoverian mares, 0.33 ± 0.04 (*μ*M*P*_*i*_/mg protein/h) (Table 4). One-way analysis of variance and Tukey's test allowed us to find significantly higher Na^+^, K^+^-ATPase activity in Hanoverian stallions in comparison to Ardenian stallions at *P* < .001. While the difference between Wielkopolska and Ardenian stallions was significant at *P* < .01. A significantly higher erythrocyte Na^+^, K^+^-ATPase activity was found in Hanoverian mares in comparison to Wielkopolska mares at *P* < .05, while the difference between Wielkopolska and Ardenian mares was significant at *P* < .001.

Correlation coefficients (*r*) concerning intra-group relationships analysis between Na^+^, K^+^-ATPase activity and ATP, ADP, AMP, TAN concentrations and AEC values in the erythrocytes of the examined horse breeds are presented in Table 5. Positive significant relationship was found between ATP erythrocyte concentration and Na^+^, K^+^-ATPase activity of erythrocytes in the groups of Wilekopolska (*r* = 0.65) and Ardenian stallions (*r* = 0.85). Moreover, significant correlation between AMP concentration and ATPase activity was found in the stallions of all the examined breeds: respectively, Wielkopolska breed *r* = 0.66, Ardenian breed *r* = 0.66, Hanoverian breed *r* = 0.53. In Hanoverian horses no significant correlations between ADP concentration and Na^+^, K^+^-ATPase activity was found. Such correlation was found in the Ardenian horses (*r* = 0.65). In Wielkopolska and Ardenian stallions a significant correlation was found between adenine nucleotides pool (TAN) and ATPase activity. Coefficient *r* was respectively 0.76 for Wielkopolska breed and 0.85 for Ardenian breed. We found significant correlation between ADP concentration and ATPase activity in the group of Wielkopolska (*r* = 0.77) and Ardenian (*r* = 0.62) mares. Significant correlations between adenylate energy charge (AEC) and Na^+^, K^+^-ATPase activity values were not found for the examine breeds (Table 5).

## 5. Discussion

The history of horses' evolution goes back 40-million years. The representatives of horses appeared on Earth millions of years before *Homo erectus*. Horse's ancestor was *Hyracotherium (Eohippus)*, which lived at the area of present Northern America. Its rear limbs had 3 digits, while the anterior limbs had 4 digits. During the evolution process many indirect forms arose (such as *Mesohippus, Merychippus i Philohippus*). 

 The modern horse is the result of a long-lasting evolution process which resulted from the natural adaptation of the animals to the environment. The appearance of the horse on steppes and the necessity of grass feeding determined the adaptation direction for millions of years. Extensive empty spaces did not provide the horse with an appropriate shelter that was why it had to learn to escape its enemy quickly and efficiently. The effects of which may be seen in the adaptation of the motor system to the fast run and unusual resistance of the organism to hypoxia and high acidity resulting from the intensive run. The above is possible due to an immense concentration of the erythrocytes in the spleen which are ready for use the moment there is a necessity to provide muscles with the oxygen. 

 Moreover multigenerational selection of horses by man for different usage purposes has brought into existence many horse breeds different not only in structure, size and beautiful appearance, but also in the rate of cell metabolism. 

The initial material for modern horse breeding of Wielkopolska horses were local, primitive forest horses, which were at first crossbred with heavy horses and then improved by oriental (Persian and Arabic) blood horses as well as by the Thoroughbred horses. Since the XVIII century the breed was created mostly by Trakehner and other horses from eastern Europe, and only partially by Hanoverian and other German half-breed horses. The selection of Wielkopolska breed was leading to forming a horse of a high weight, appropriate and efficient movement and gentle nature—suitable for drawing. Modern Wielkopolska horses are mainly functional animals, with the majority of riding characteristics. 

Hanoverian horses are recognized as the best sport horses bred in Germany. They appeared at the beginning of the XVIII century. Their appearance was greatly influenced by a state owned stud in Celle, where, at the beginning, Holsteiner stallions were used as sires. After the Second World War the Hanoverian horse breeding led to the creation of sport horse and for that reason it was crossbred with Trakehners (resistance improvement) and with Thoroughbred horses (the improvement of courage and movement). Modern Hanoverian horses are characterized by high strength, appropriate movement, gentle nature and good boniness. The Hanoverian horse is good for competitive sport, especially for dressage and horse jumping. Although the Hanoverian is not a sprinter due to being a functional animal; thanks to its strength and efficient movement it is as effective as other breeds when in a run. 

The Ardenian breed derived from the ancient horses coming from Solutré. In France the following types are recognized: French Arden (called Eastern); *trait du nord* (Northen Arden) i *auxois*. The functional characteristics of the Ardenian horses were influenced by Belgian horses, small Brabansons, Percherons, Boulonnais and Norman horses. This obedient, calm, massive, strong and muscular horses are perfect for physical works. All the Ardenian horses are mainly used for meat. Belgian, Luxemburg and Swedish Ardens also belong of the group of the Ardenian horses. 

Comparing individual average adenine nucleotides concentration values in red blood cells of the examined horses it may be assumed that the ATP concentration is several dozen times higher than the AMP concentration, and ADP is of average value. Relationships among ATP, ADP and AMP concentrations determine the adenylate energy charge (AEC) value, which under physiological conditions, is always 0.8-0.9, regardless of cell type and generic features [23].

Based on the literature high interspecies changeability of the erythrocytic ATP concentration may be assumed. Approximate ATP, ADP, and AMP contents in chosen mammals are presented in Table 6. 

The obtained research results explicitly suggest that among the examined mammals, horses, regardless of breed, have relatively low physiological ATP concentration in the erythrocytes, which was also confirmed by other authors [24, 25]. Despite low ATP erythrocytic concentration, energy charge (AEC) value was relatively constant and comprised within the 0.89–0.92 range. Adenine nucleotides pools (TAN) of the examined horses, on the other hand, varied depending on breed and sex. 

The relatively low ATP concentration of erythrocytes found in horses compared to other animal species may be the result of the erythrocytes size, the diameter of which is about 5 *μ*m [26]. Therefore, the hypothesis that a connection may exist between small erythrocyte size, low ATP concentration and lively breathing of the organism may find confirmation in our data. 

The highest adenine nucleotides pool (TAN) was found in stallions and mares of breathing constitution type horses of Wielkopolska breed, and the lowest in digestive constitution type horses of Ardenian breed. The ATP concentration which decides about adenylate pool in erythrocytes was significantly higher in Wielkopolska horses in comparison to Hanoverian and Ardenian breeds. Leyko et al. [27] found that in the erythrocytes adenine nucleotides concentration, TAN values and AEC are determined genetically, which was also proven by our study. The changes of the ATP concentration in the erythrocytes of the examined horses may possibly be connected with the changes of the hematological indicators' values of the examined animals since the highest amount of red blood cells and hemoglobin were found in Wielkopolska horses (stallions: RBC—8.31 × 10^12^/L ± 0.39, Hb—98 g/L ± 9.1; mares: RBC—8.27 × 10^12^/L ± 0.61, Hb—92 g/L ± 7.5), najniższą u rasy ardeńskiej (stallions: RBC—7.71 × 10^12^/L ± 0.97, Hb—77 g/L ± 2.8 mares: RBC—7.53 × 10^12^/L ± 0.81, Hb—73 g/L ± 7.1). Genetically determined higher amount of red blood cells is favorable for a better adaptation of the horse to a physical effort of a high-speed type and for more effective gas exchange connected with an adaptation to a long run [11, 27, 28]. The observed differences were formed during a long, multigenerational selection of horses. They prove better predispositions and adaptation to a high-speed effort in breathing constitution type horses (Wielkopolska and Hanoverian breeds) compared to digestive constitution type (Ardenian breed) which is rather predisposed to endurance exertion.

Harvey and Kaneko [28] indicate that in physiological conditions there are certain generic differences in the utilization of glucose in the process of glycolysis. Horse erythrocytes have a higher hexokinase activity (HK, EC 2.7.1.1) in comparison to other animals which may influence the rate of metabolic utilization of glucose. However, the glucose utilization rate in horse erythrocytes is half the rate of humans and dogs, and 2/3 in comparison to cat's erythrocytes [28]. Still as much as 13% of glucose used by horses is metabolized in the pentose phosphate pathway, while humans, cats and dogs only use half (about 6%). Glucose-6-phosphate (G-6-P), created in the reaction catalyzed by HK, acting as an allosteric inhibitor of the enzyme, may reduce glucose utilization in the process of glycolysis [29]. It was proven that G-6-P regulation depends on intracellular pH. Optimal pH value for the glycolysis process is 8.2, and for pentose phosphate pathway: 7.6 [28]. 

A relatively high glucose utilization in the pentose phosphate pathway [28] and a low glucose utilization in the process of glycolysis in horses may result in a relatively low ATP concentration in horses in comparison to other species [5]. Breathing constitution type horses' erythrocytes (Wielkopolska and Hanoverian breeds) may be subjected to more intensive glucose changes and more effective energetic metabolism of the erythrocytes in comparison to digestive constitution type horses (Ardenian breed). This is suggested by our data, which have proven the existence of higher ATP concentration, higher adenine nucleotides pools (TAN) and a significantly higher adenylate energy charge (AEC), which may make breathing constitution type horses more suitable for long distance runs [30]. Moreover, higher erythrocytic ATP concentration and higher TAN values of breathing constitution type horses may be the result of a higher adenylate kinase activity (AK, EC 2.7.4.3) catalyzing the reaction 2 ADP = ATP + AMP. In the short term this mechanism protects cells against an excessive drop of ATP concentration and nucleotides pool [31].

 Some mammals (a rat, a mouse, a rabbit, a human, a dog, a pig) have significant amounts of 2,3-BPG in the erythrocytes compared to a sheep and a cow [11]. This metabolite is responsible for intraerythrocytic regulatory mechanism of oxygen releasing from the blood to the cells. It also participates in the regulation of RBC metabolism [4, 32, 33]. The increase of 2,3-BPG amount in blood indicates the decrease of hemoglobin's affinity for oxygen and the increase of *P*_50_ value (partial oxygen pressure at which 50%  of the hemoglobin is saturated with oxygen). This leads to the increased ability for oxygen delivery to the tissues and more intensive metabolism. The amount of glucose metabolized to create 2,3-BPG in human erythrocytes under physiological conditions was estimated for 19% and it depends on ADP concentration in RBC [34]. With the decrease of ADP concentration in the erythrocytes 2,3-BPG concentration increases, if, however, the ADP concentration compared to ATP is high, 2,3-BPG decrease may be observed. The regulatory mechanism of 2,3-BPG concentration by ADP results from the fact that ADP and 2,3-BPG are the reagents in the reaction of phosphoglicerine kinase (PGK, EC 2.7.2.3) and the changes of their concentrations may influence the content of 1,3-BPG [33]. 

Keeping higher 2,3-BPG concentration in the erythrocytes is energetically expensive but it allows for a proper regulation of hemoglobin's affinity for oxygen to the speed of the metabolism. It is physiologically favorable as lower oxygen affinity (higher *P*_50_ values) allows for easier oxygen delivery to the tissues in animals characterized by more intensive metabolism. 

Sex does not have a significant influence on adenine nucleotides concentration (ATP, ADP, AMP) in red blood cells of the examined horses, however, stallions have slightly higher values in comparison to mares.

A positive correlation between ATP, ADP, AMP, and TAN concentrations and Na^+^, K^+^-ATPase activity of the erythrocytes was found in Wielkopolska and Ardenian horses, which was not confirmed in Hanoverian breed. Na^+^, K^+^-ATPase activity depends on the breed. The highest enzyme activity was found in Wielkopolska breed, and the lowest in Ardenian horses. The interbreed differences in the enzyme activity were also observed in humans [35]. 

Na^+^, K^+^-ATPase activity regulation is influenced by many endogenous factors [36]. The enzyme activity depends on the saturation degree of the substrates binding sites and on the cytoplasmatic products of the enzymatic reaction—ADP and *P*_*i*_ concentration [37]. 

The inhibit influence of ADP on the enzyme activity based on competition between ADP and ATP for enzyme protein binding sites. The effect of ADP and Na^+^, K^+^-ATPase binding is lowered when the ATP concentration is higher [37]. Both in vitro and in vivo researches have shown that the K^+^ concentration increase in the erythrocytes results in a higher Na^+^, K^+^-ATPase activity. However, lowering of the K^+^ concentration in the solution of the cell, or partial saturation of Na^+^, K^+^-ATPase binding sites, results in the restrain of the enzyme activity [37, 38]. Na^+^, K^+^-ATPase activity of the membrane cell is significantly influenced by pH (the highest activity at pH = 9.5, the lowest activity at pH = 6.0). Enzyme activity also depends on Ca^2+^ concentration. Ca^2+^ may compete with K^+^ or Mg^+^ (reaction cofactors) for enzyme receptor site [39]. We found lower Na^+^, K^+^-ATPase activity in the examined mares in comparison to stallions, with a simultaneous increase of K^+^ concentration and decrease of Na^+^ concentration in serum. It may be related to the hormonal state of the mares' organisms [40].

## Figures and Tables

**Table 1:** Concentration of adenine nucleotides: ATP, ADP, AMP (*μ*M), total adenylate nucleotide pool (TAN) and values of the adenylate energy charge (AEC) in erythrocytes of stallions Wielkopolska breed (W), Hanoverian breed (H), Ardenian breed (A) Legend: $\overset{®}{x}$-mean; ±SD—standard deviation; **-significant difference (*P* < .01); *-significant difference (*P* < .05), *n*-group size.

Group	*n*		ATP	ADP	AMP	TAN	AEC	RBC [10^12^/L]	Hb [g/L]
			*μ*M_*Μ*RBC_						
W	8	*x*	178.3	28.3	5.5	212.1	0.91	8.31	98
		±SD	12.5	3.8	1.6	14.7	0.013	0.39	9.1
H	8	*x*	155.0	24.9	4.3	184.2	0.91	8.01	91
		±SD	11.3	4.2	0.8	13.0	0.012	0.76	3.8
A	10	*x*	148.7	29.5	5.5	183.8	0.89	7.71	77
		±SD	12.9	2.8	0.5	15.3	0.006	0.97	2.8

Statistical significance			W, A**	A, H*	A, H*	W, H*	H, A**	W, A*	W, A*
of the group differences			W, H*			W, A*	W, A*		A, H*

**Table 2:** Concentration of adenine nucleotides: ATP, ADP, AMP (*μ*M), total adenylate nucleotide pool (TAN) and values of the adenylate energy charge (AEC) in erythrocytes of mares Wielkopolska breed (w), Hanoverian breed (h), Ardenian breed (a) Legend: $\overset{®}{x}$-mean; ±SD*—*standard deviation; **- significant difference (*P *< .01); *- significant difference (*P *< .05), n-group size.

Group	*n*		ATP	ADP	AMP	TAN	AEC	RBC [10^12^/L]	Hb [g/L]
			*μ*M_*Μ*RBC_						
w	8	*x*	175.4	27.8	6.1	209.3	0.90	8.27	92
		±SD	9.6	3.2	1.1	10.7	0.004	0.61	7.5
h	10	*x*	150.4	24.0	4.0	178.5	0.91	8.05	88
		±SD	21.5	1.8	0.5	21.0	0.013	0.77	2.2
a	10	*x*	147.3	28.0	5.4	180.7	0.89	7.53	73
		±SD	21.2	2.9	0.6	23.0	0.010	0.81	7.1

Statistical significance			w, a*	h, a**	h, a**	w, h*	h, a**	w, a*	w, a*
of the group differences			w, h*	w, h*	w, h**	w, a*			h, a*

**Table 3:** Correlation coefficient (*r*) between ATP concentration and total adenylate nucleotide pool (TAN) and values of the adenylate energy charge (AEC) in erythrocytes of stallions and mares Wielkopolska breed (W,w), Hanoverian breed (H,h), Ardenian breed (A, a); *-significant difference (*P* < .05).

	ATP					
	W	H	A	w	h	a
TAN (*μ*M_*Μ*RBC_)	0.93	0.94	0.99	0.96	1.00	0.99
AEC	0.65	0.97	0.76	0.67	0.97	0.77

**Table 4:** The Na^+^, K^+^-ATPase activities of the erythrocytes and Na^+^: K^+^ions ratio in blood serum of stallions and mares Wielkopolska breed (W, w), Hanoverian breed (H, h), Ardenian breed (A, a).

Group	*n*		Na^+^, K^+^-ATPase	Na^+^ : K^+^
			*μ*mol*P*_*i*_/mg protein/h	
W	8	*x*	0.34	32.30
		±SD	0.06	
H	8	*x*	0.34	34.80
		±SD	0.01	
A	10	*x*	0.22	37.70
		±SD	0.05	
w	8	*x*	0.28	34.20
		±SD	0.02	
h	10	*x*	0.33	34.70
		±SD	0.04	
a	10	*x*	0.19	39.20
		±SD	0.02	

**Table 5:** Correlation coefficient (*r*) between adenine nucleotides concentration (*μ*M), total adenylate nucleotide pool (TAN), values of the adenylate energy charge (AEC) in erythrocytes and the Na^+^, K^+^-ATPase activities of stallions and mares Wielkopolska breed (W, w), Hanoverian breed (H, h), Ardenian breed (A, a); *-significant difference (*P* < .05).

Dependent variable		Independent variable									
		ATP		ADP		AMP		TAN		AEC	
		*μ*M_*Μ*RBC_									
Na^+^, K^+^-ATPase *μ*mol*P*_*i*_/mg protein/h		stallions	mares	stallions	Mares	stallions	mares	stallions	mares	stallions	mares
	W, w	0.65*	0.35	0.49	0.76*	0.66*	0.56*	0.76*	0.48	−0.37	−0.09
	H, h	0.10	0.40	0.43	0.20	0.53*	0.01	0.26	0.43	−0.46	0.32
	A, a	0.85*	0.47	0.65*	0.66*	0.66*	0.36	0.85*	0.52*	0.23	0.52*

**Table 6:** Concentration of adenine nucleotides: ATP, ADP, AMP (*μ*M) in erythrocytes of chosen animal species (the own study based on the references [11]^B^, [12]^C^, [14]^D^, [25]^A^).

Species of mammals	human	Pig	horse	goat	dog
ATP [*μ*M_*Μ*RBC_]	1650^A^	2220^A^	120^A^	710^A^	590^A^
	1438^B^		184.9^D^		639^B^
	1519^C^				
ADP [*μ*M_*Μ*RBC_]	216^B^		28.8^D^		138^B^
	171^C^				
AMP [*μ*M_*Μ*RBC_]	21^B^		5.7^D^		40^B^
	12^C^				

## Abbreviations

AK: adenylate kinase (EC. 2.7.4.3)
ADP: adenosine-5'-diphosphate
AEC: adenylate energy charge
AMP: adenosine-5'-monophosphate
ATP: adenosine-5'-triphosphate
1,3-BPG: 1,3-biphosphoglycerate
2,3-BPG: 2,3-biphosphoglycerate
G-6-P: glucose-6-phosphate
Hb: hemoglobin
HK: hexokinase (EC 2.7.1.1)
P_50_: partial oxygen pressure at which 50% of the hemoglobin is saturated with oxygen
PGK: phosphoglicerate kinase (EC 2.7.2.3)
TAN: total adenine nucleotides, total nucleotide pool
